# Reliability and robustness of a novel preclinical torsional wave-based device for stiffness evaluation

**DOI:** 10.1038/s41598-024-66661-2

**Published:** 2024-07-16

**Authors:** Alberto García, Pablo Diez, Guillermo Rus, Antonio Callejas, Jorge Torres

**Affiliations:** 1 Innitius, Derio, 48160 Spain; 2 Ultrasonics Lab (TEP-959), Department of Structural Mechanics, University of Granada, Granada, 18071 Spain; 3 TEC-12 Group, Instituto de Investigación Biosanitaria, ibs., Granada, 18001 Spain; 4 LabTAU, INSERM, Centre Léon Bérard, Université Claude Bernard Lyon 1, Lyon, F-69003 France

**Keywords:** Torsional Wave Elastography, Shear Wave Elastography, Mechanical biomarkers, Tissue biomarkers, Cervix, Intraclass correlation coefficient, Biomedical engineering, Diagnostic markers

## Abstract

In this work, we present a novel preclinical device utilizing Torsional Wave Elastography (TWE). It comprises a rotational actuator element and a piezoceramic receiver ring circumferentially aligned. Both allow the transmission of shear waves that interact with the tissue before being received. Our main objective is to demonstrate and characterize the reliability, robustness, and accuracy of the device for characterizing the stiffness of elastic materials and soft tissues. Experimental tests are performed using two sets of tissue mimicking phantoms. The first set consists of calibrated CIRS gels with known stiffness value, while the second test uses non-calibrated manufactured phantoms. Our experimental observations show that the proposed device consistently and repeatably quantifies the stiffness of elastic materials with high accuracy. Furthermore, comparison with established techniques demonstrates a very high correlation (> 95%), supporting the potential medical application of this technology. The results obtained pave the way for a cross-sectional study aiming to investigate the correlation between gestational age and cervical elastic properties during pregnancy.

## Introduction

Torsional waves can be used to characterize the elastic properties of materials, in particular stiffness of soft tissues^1^. They have been used in many applications such as guided waves in nondestructive testing of pipes^2,3^, in liquids inserting a bar in the fluid where waves are propagated to measure density^4,5^, liquid level^6^, temperature^6^, or viscosity^5^. For clinical application, elastography has been used as a medical imaging method that assesses soft tissue stiffness by inducing vibrations in the tissue and analysing its propagation and deformation^7^. This diagnostic approach aids in identifying conditions like liver fibrosis and breast cancer^8^. Being non-invasive and painless, elastography is gaining prominence in medical diagnostics and therapeutic interventions. Despite its diverse applications, elastography presents ongoing challenges, such us: standardization of techniques, validation of quantitative measures, and integration with other imaging modalities are actively pursued^7^. There also exists a special interest in efficiency enhancements of sensor designs^9–14^. Addressing these challenges, alternative dynamic techniques based on torsional vibrations have been explored for characterizing the viscoelastic properties of soft tissues. For instance, Valtorta et al.^9^ proposed a method utilizing a torsional resonator to measure the complex shear modulus of soft biological tissues, demonstrating feasibility through in vitro experiments on bovine and porcine liver. Henniet al.^10^ introduced a shear wave-induced resonance technique for dynamic ultrasound elastography, validated in vivo on a breast fibroadenoma. Accordingly, the motivation of this study began with the aim of designing an optimized torsional wave sensor version focused on clinical applications to obtain high levels of sensitivity in mechanical identification of soft tissue. The proposed torsional wave probe (Innitius, Spain) is based on the devices developed by the Ultrasonics Lab, whose initial objective was to estimate cervical stiffness as a predictor of preterm delivery^11–15^. This sensor can measure the shear wave speed in tissue mimicking phantoms and soft tissues. A part of the computational design of the transducer was previously described in detail^15^. These studies have laid the groundwork by characterizing the cervical tissue and optimizing the computational design of the torsional transducer, which have allowed the development of the device used in this study. This is, therefore, the result of the research work carried out which has resulted in a device dedicated to the measurement of cervical consistency using torsional waves and which has motivated this study to check its reliability and robustness in the measurement of consistency and to compare its performance against established technologies on the market.

The cervix, a vital muscular component situated at the lower end of the uterus in the female reproductive system, assumes a pivotal role during gestation^16^. Functioning as a gatekeeper, the cervix provides essential support to the developing fetus within the uterus, safeguarding it from external hazards throughout pregnancy^17,18^. The mechanical transformation during gestation consists in a decrease of stiffness of the cervix^17,19,20^. The dynamic nature of the cervix undergoes profound biological transformations from the moment of conception until childbirth, triggered by intricate mechanical and chemical processes^20,21^. Notably, these transformations exert a significant impact on the physiological aspects of cervical tissue, leading to alterations in its mechanical properties^16,22^.

In this study, we propose a novel preclinical device employing Torsional Wave Elastography (TWE) to measure the stiffness of elastic materials,whose ultimate goal will be to measure the stiffness of the cervix. This technique utilizes a transvaginal probe, establishing contact with the medium to transmit and receive torsional waves propagated through the tissue. The aim of this technique is to differentiate between true and false threats of preterm labor, using cervical tissue stiffness obtained with the device and other clinical variables of the patient. As a non-invasive, in-vivo diagnostic technology, the Innitius TWE device employs torsional waves to precisely quantify the mechanical properties of cervical tissue, specifically the shear modulus (expressed in kPa), indicative of the resistance opposing shear deformations, commonly referred to as consistency or stiffness. The objective of this work is to evaluate the reliability and feasibility of the novel TWE device and carry out a validation using established Shear Wave Elastography (SWE). We present a detailed description of the device structure, emphasizing its optimal design. The device incorporates three main elements that allow it to be used in a clinical setting: an automatic protective membrane placement system, a force system to monitor the force applied to the tissue in real time, and a camera to visualize the tip of the device in real time. Subsequently, the experimental setup and methodology are outlined, incorporating tests on tissue-mimicking phantoms to confirm the independence of measurements from user, device, and phantom surface variations.

## Material and methods

### Description of the TWE device

**Figure 1:**
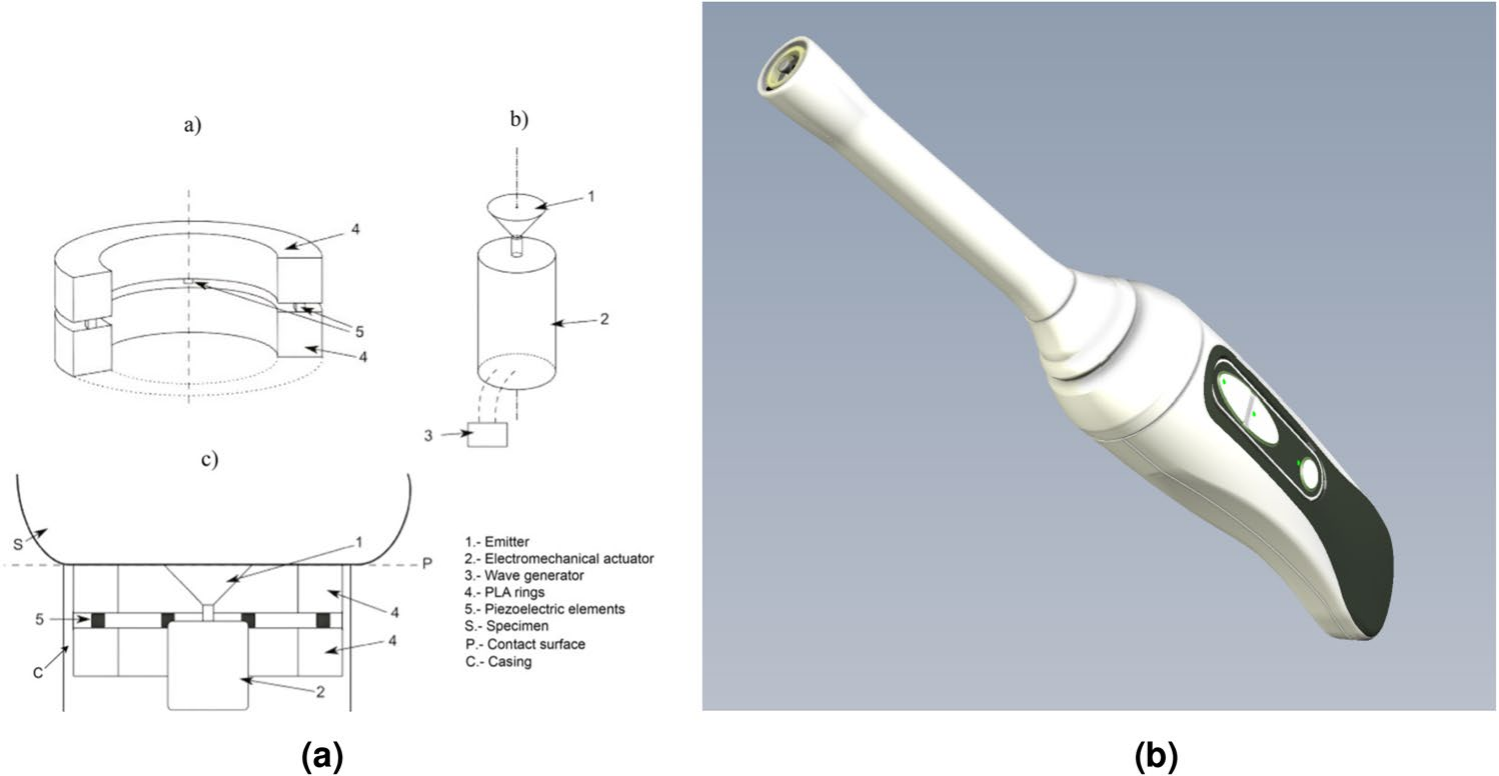
(**a**) Elements of TWE technology (**a**) shows a receiver consisting of 2 rings of biocompatible material and the piezoelectric sensors. (**b**) Emitter consisting of an electromechanical actuator, a specimen contact element, and (**c**) configuration of the device where emitter and receiver share the same axis and are located in the same plane of contact with the specimen. (**b**) shows the probe of Innitius, with the torsional wave transducer into a probe for measuring cervix stiffness.

The torsional wave technology implemented in the device, as illustrated in Fig. 1a, comprises three essential components: an emitter, a receiver, and a casing that securely houses both elements. Its design is based on previous research prototypes^11,12,23^. The emitter, responsible for transmitting the torsional waves, contacts the medium through a 3D-printed disk (Fig. 1a). This disk undergoes rotational movement facilitated by an electromechanical actuator. Electric excitation of the actuator is achieved through a wave generator, where parameters such as frequency, voltage, and working cycle are precisely controlled. Both the probe and the wave generator are controlled by a standalone microcontroller that manages the device in real-time and allows for portable use. The receiver, depicted in Fig. 1a, is constructed with two rings of biocompatible non-conductive material, two PCB connection track rings that allow the positive and negative poles of the sensors to be connected, and four piezoelectric sensors arranged in a cross position to ensure equidistance. These rings contribute to inertia reduction and lower the resonant frequency, while the array of transversely polarized piezoceramic elements transforms mechanical vibrations into an electric signal^11^.

To make this technology applicable to real envirorment, the components are integrated into a preclinical probe (Fig. 1b). This device facilitates the acquisition of torsional waves in the cervical tissue. The fundamental concept revolves around the necessity for a significant reduction in cervix consistency to facilitate vaginal delivery. The device is able to directly evaluate the critical change in stiffness required for successful delivery^24^. The reliability and repeatability of the technology are ensured through several subsystems, with the main components described in more detail in the following sections.

#### Tip of the probe

**Figure 2:**
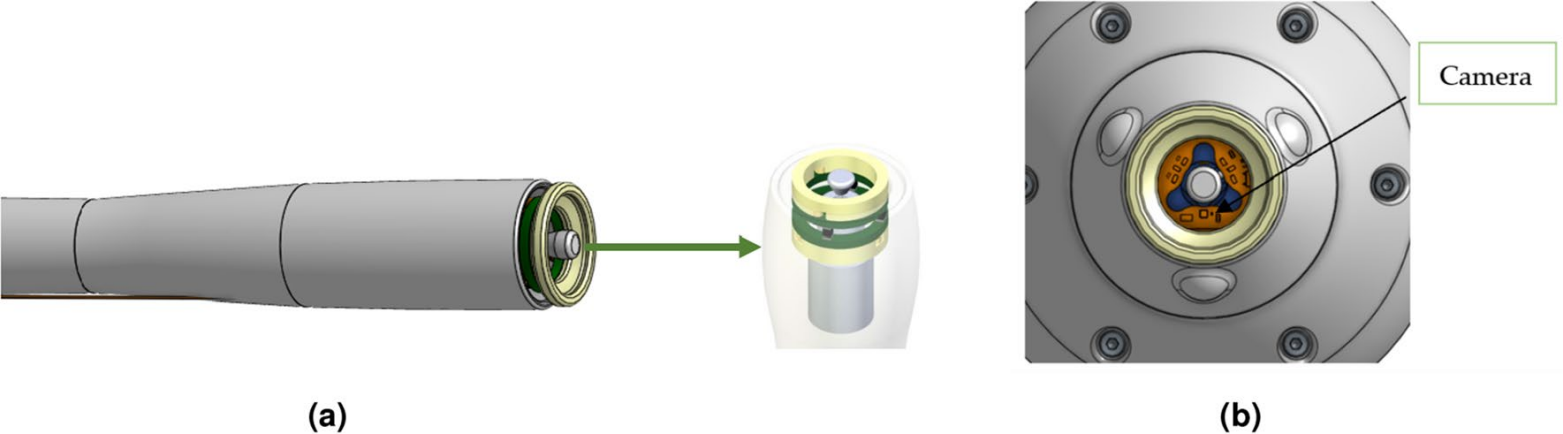
Tip of the probe principal elements. (**a**) Disposition of set emitter-receiver inside the TW probe. (**b**) Camera location on the tip of the TW device.

As explained in the previous section, the torsional wave technology employed in the Innitius probe requires the emitter centered inside the receiver ring. To guarantee this action, a motor support part is used to hold the emitter.When this support piece does not perform its function correctly, the relative transmitter-receiver position is not concentric and therefore the distances are not equidistant, which leads to variability in the received signals and therefore to an error in the accuracy of the system when obtaining the stiffness (see Fig. 2a).

The TW Probe of Innitius, as a contact-based methodology, needs to ensure accurate measurements by positioning the specimen between the emitter and receiver. Only the region of the medium situated between the emitter-receiver air gap, through which torsional waves propagate.To make it easier for the user to position the device, a display system has been implemented, comprising the camera and display components, serves the purpose of enabling real-time visualization of the probe tip. This functionality allows the user to discern the support region captured by the camera. It is essential to note that while the system does not explicitly indicate or provide feedback on the proper positioning of the probe, it acts as a valuable support tool. Figure 2b shows the location of the camera on the torsional wave device.

#### Force system

**Figure 3:**
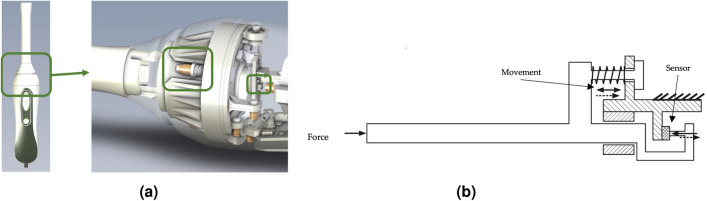
Description of the force system of the TW device. (**a**) Components of the force system. (**b**) Movement of the force sensor of the TW probe.

The device includes a force measurement system, (Fig. 3), which is designed to determine the force applied by the user on the medium being measured, expressed in g/cm2. This system serves the critical purpose of precisely quantifying the applied force, providing valuable feedback to the user. The feedback mechanism enables users to make necessary adjustments to ensure the applied force falls within an optimal range, thereby guaranteeing the consistency of results. This iterative process of feedback and adjustment enhances the accuracy and reliability. Additionally, controlling pressure is crucial in elastography to avoid non-linearities that compromise the accuracy of stiffness measurements^1^.

Force system is located in the middle part of the device, green box of the left image, joining the handle and the tip (Fig. 3a). It detects the force applied with the entire tip of the device on the surface, using a bearing system to allow the tip to slide along the longitudinal axis towards the handle for distances of less than 1 mm, and presses on a force sensor located at the bottom of the system, shown on the green boxes on the right picture. A set of springs allows the return point to return to the system if no force is applied. Figure 3b shows a schematic of the movement and displacement of the system when force is applied to the tip, and how the different components work to apply the force directly to the sensor so that it can be measured.

#### Vacuum system

**Figure 4:**
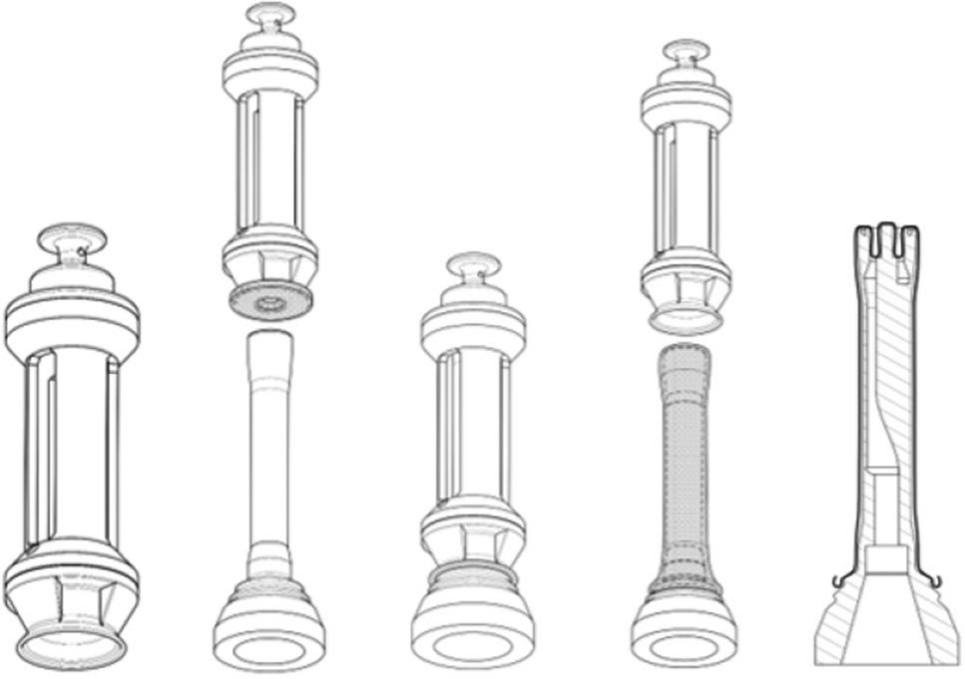
Membrane placement procedure. The sequence from left to right illustrates the membrane placement process. Initially, the membrane placement tool is shown with the condom mounted on it. Next, the tool, with the condom secured, is positioned onto the probe tip. The third image depicts the final positioning of the tool to ensure the condom is correctly placed. Following this, with the vacuum activated, the membrane is shown positioned over the tip as the tool is removed. The final image shows the condom properly placed on the probe tip.

To maintain a sterile environment and prevent cross-contamination between patients and the device, the use of a single-use membrane is mandatory. The generation and reception of torsional waves needs a specific and repeatable placement system to avoid any interference with the device’s functionality due to the membrane, and consequently to the stiffness result. For this purpose, a comprehensive solution and a repeatable procedure for placement have been designed, as shown in Fig. 4.

### Time of flight calculation for stiffness measurement

One of the main advantages of dynamic elastography with respect to other techniques is the direct relationship between wave velocity and mechanical properties, eliminating the need for complex stress distribution calculations. In an elastic, isotropic, and incompressible medium, the stiffness ($\mu$) is obtained as $\mu$= $\rho$ $c_{s}^{2}$ where $\rho$ is the density of the medium, with an assumed value of 1000 kg/$m^{3}$ and $c_{s}$ is the torsional wave velocity.

**Figure 5:**
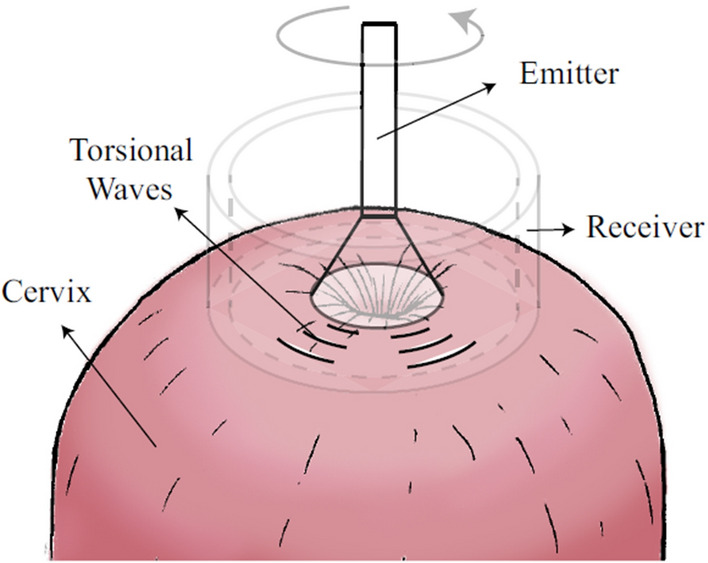
Schematic diagram of the propagation of the torsional waves across the cervix tissue. Adapted from^12^.

The placement of the device in a soft tissue sample can be seen in Fig. 5. The method employs a time-of-flight (TOF) approach for estimating the velocity. While $c_{s}$ ideally could be directly calculated from the time of flight, the time of flight measured directly from the sensor signal is not accurate due to delays associated with various factors^12,24^.

These internal delays are inherent to the measurement configuration and require prior estimation. Assuming that the delays depend only on the material and the frequency of excitation, time-of-flight measurements at varying emitter-receiver distances can be extrapolated to a zero distance, to obtain a non-zero value representing the delay. These effects are consolidated into a single delay time factor, resulting in the velocity calculation using the formula:

1 $$\begin{aligned} \begin{aligned} c_s=\frac{Distance}{TOF-delay} \end{aligned} \end{aligned}$$

**Figure 6:**
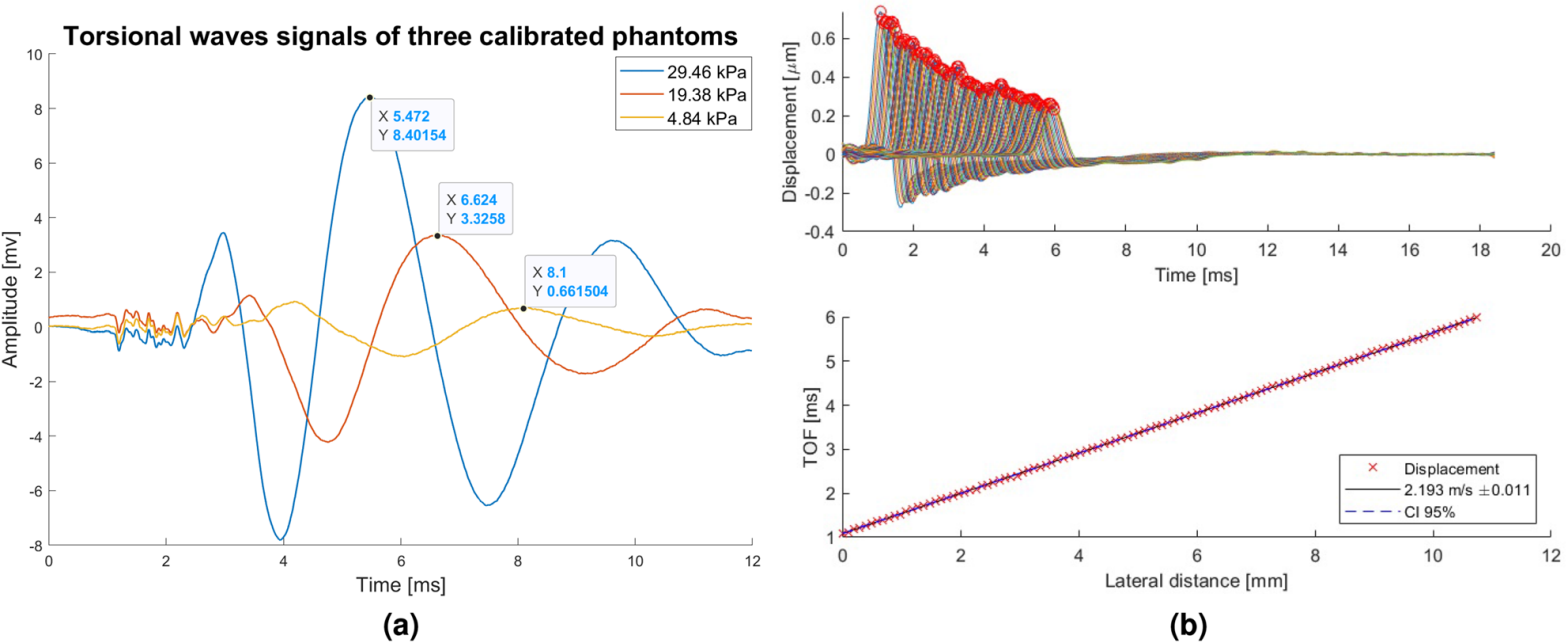
Signals comparison between techniques. (**a**) Torsional waves signals received from three different phantoms. (**b**) Analysis image in Verasonics (SWE) on CIRS phantom.

For the calculation of the velocity, therefore, the dependent variable of the material on which the measurement is made is the time of flight (TOF), since the emitter-receiver distance and the delay inherent to the device are kept constant^12^. An example of three different received torsional signals is shown in Figure 6a.

### Validation against SWE

To validate the estimated values from the Innitius Torsional Wave device, SWE was conducted using a Verasonics research system (Vantage 256, Verasonics Inc., Redmond, WA, USA). To remotely induce shear waves, an Acoustic Radiation Force push of 1000 cycles was applied to the medium utilizing an L11-5v linear probe. The central frequency employed was 7.6 MHz, and the focal distance was set at 25 mm. Subsequently, ultrafast imaging was performed, utilizing plane waves with an acquisition rate of 12.5 kHz. To mitigate the impact of random noise, 10 consecutive frames were averaged immediately after acquisition. Group shear wave velocity is reconstructed by tracking the motion of multiple points^25^. This methodology initiates by identifying the arrival time of shear waves at various propagation distances, utilizing the time-to-peak (TTP) feature. Subsequently, a linear regression analysis is employed to establish the relationship between TTP and the propagating distance. See analysis in Figure 6b.

### Materials description

**Figure 7:**
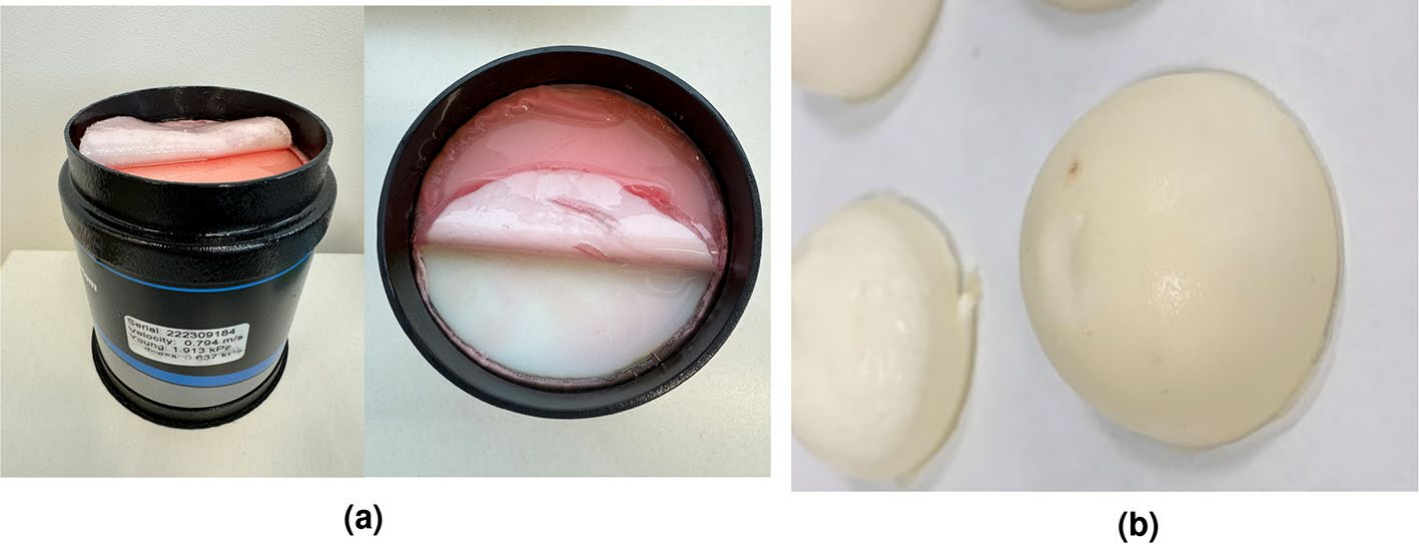
Phantoms use for the study. CIRS (**a**) and laboratory manufactured (**b**) phantoms. (**a**) CIRS samples. On the left, with the protective oil and on the right with membrane removed to allow the measurement of the TW device. (**b**) Non-calibrated phantoms.

In order to assess the reliability and reproducibility of the proposed device, phantoms are employed as test subjects to simulate a range of stiffness values of real tissue. The experimental design involves three users conducting three measurements for each sample, utilizing three different devices. All measurements are conducted at room temperature (21 $^\circ$C). The obtained values are compared against those derived from the SWE technique (Verasonics), where each sample was also measured three times. Two sets of experiments are performed. The first set uses calibrated and certified phantoms. Seven phantoms (039 model, CIRS Inc., USA), each filled with Zerdine$\circledR$ formulated with different stiffness values, ranging from 2.25 to 37.33 kPa are analyzed (Fig. 7a). Shear wave velocity certification is provided with each material, with testing performed using SWE following the protocol of the Quantitative Imaging Biomarkers Alliance (QIBA) Ultrasound Shear Wave Velocity Committee.

The second set uses laboratory manufactured phantoms. For this test, seven samples with different stiffness, by varying the amount of gelatin, are manufactured. Amounts of gelatin from 7 to 25% are used, by keeping the amount of other ingredients constant. K-Sorbate is 1%, oil is 5%, and surfactant is 0.30%. To make the samples as close to reality as possible, the samples were made in semi-circular molds to simulate the anatomy of the cervix (Fig. 7b).

The steps followed to make the phantoms are described below^26^. All samples were created using a base of 250 ml of water to have the correct amount of material available for measurements. The process to be followed for the fabrication of the samples is as follows:

1. Heat 250 ml of water in the microwave
2. Add cellulose. The cellulose serves as a scatterer to facilitate the mesurement with Verasonics.
3. Gradually add the gelatin powder to the solution.
4. Place in the ultrasonic mixer for 5 min. This allows the mixture to be as homogeneous as possible.
5. Add the surfactant and continue mixing for another 5 min.
6. Add the oil with the previous solution and mix manually at a rate that minimizes the formation of air bubbles and the formation of large lumps.
7. Place in the ultrasonic mixer for 5 min. This allows the mixture to be as homogeneous as possible.
8. Allow the phantom to solidify at room temperature for 2 h before storing in the refrigerator.
9. Remove the phantom from the refrigerator and leave it at room temperature for 6 h.

### Measurement protocol

All measurements have been performed with the same experimental protocol: A total of 3 users have carried out the measurements, perform 3 measurements for each of the samples presented in the section before and using 3 different devices, for repeatability and robustness purposes. The phantoms shall be measured at room temperature (21 $^\circ$C).

### Statistical methods

To assess the concordance between the different consistency measurements several metrics have been used, being the most commonly used the Intra Class Correlation Coefficient (ICC) with 95% CI. This metric was first introduced by Fisher in 1954 as a modification of the Pearson correlation coefficient and it is, nowadays, widely used in the medical field to assess intra and inter-operator reliability^27,28^. The ICC index is used to calculate repeatability. In this case, Two-way random Intraclass Correlation Coefficient (ICC) with 95% CI and statistical significance was obtained using the Fisher’s Test and its associated p-value. The ICC values were interpreted as: ICC > 0.9: excellent reliability, 0.75 < ICC < 0.9: good reliability, 0.5 < ICC < 0.75: moderate reliability, and ICC < 0.5 poor reliability. The level of statistical significance was set at 0.05^29,30^.

Bland Altman plots have also been obtained to evaluate the similarities between different devices and operators^31^. These plots, also known as difference plots, are a powerful graphical tool for comparing two measurement techniques and evaluating the agreement between two sets of data.

Also, the concordance correlation coefficient (CCC) was used. The CCC measures the agreement between two variables, e.g., to evaluate reproducibility or for inter-operator reliability.

Finally, a comparison between the gold standard used technique (SWE measurements) and Innitius TW device stiffness calculation using torsional waves has been done, by contrasting the values obtained by these two techniques qualitatively due to the different nature of the techniques. All of these calculations have been performed using python libraries scipy, scikitlearn and pingouin.

## Results

### Calibrated phantoms

As mentioned above, the test structure consists of 3 operators and 3 devices, with which each user will perform measurements on the different samples. The devices used in the test will be coded as: Device 1, Device 2 and Device 3. Tables 1, 2 and 3 report the results in the form of mean ± standard deviation and repeatability, for the Device 1, 2, and 3, respectively.

**Table 1:** Results on calibrated phantoms of Device 1 for the three users.

Device 1							
Gel stiffnes (kPa)	2.24	2.83	4.84	9.12	9.55	19.39	29.46
Operator 1($\mu \pm \sigma$)	1.94 ± 0.36	2.58 ± 0.21	5.45 ± 0.47	9.10 ± 0.16	10.29 ± 0.20	19.38 ± 1.35	31.14 ± 0.64
Repeatibility	81.82%	91.64%	91.19%	98.17%	98.05%	92.99%	97.94%
Deviation	9%	6%	9%	0%	5%	0%	4%
Operator 2 ($\mu \pm \sigma$)	1.95 ± 0.05	2.91 ± 0.17	5.24 ± 0.20	9.29 ± 0.12	9.38 ± 0.13	18.43 ± 0.48	25.78 ± 0.59
Repeatibility	97.36%	93.97%	96.1%	98.7%	98.61%	97.35%	97.69%
Deviation	9%	2%	6%	1%	1%	3%	9%
Operator 3 ($\mu \pm \sigma$)	2.53 ± 0.15	2.73 ± 0.26	5.51 ± 0.08	8.53 ± 0.78	10.04 ± 0.57	16.86 ± 1.12	26.48 ± 1.91
Repeatibility	99.40%	90.21%	98.51%	90.85%	94.31%	93.37%	92.81%
Deviation	9%	2%	10%	5%	5%	9%	7%

**Table 2:** Results on calibrated phantoms of Device 2 for the three users.

Device 2							
Gel stiffnes (kPa)	2.24	2.83	4.84	9.12	9.55	19.39	29.46
Operator 1($\mu \pm \sigma$)	2.25 ± 0.17	2.79 ± 0.02	4.92 ± 0.04	9.30 ± 0.16	9.50 ± 0.06	18.12 ± 1.68	27.48 ± 1.61
Repeatibility	92.43%	99.23%	99.22%	98.27%	99.36%	90.68%	94.14%
Deviation	1%	1%	1%	1%	0%	5%	5%
Operator 2 ($\mu \pm \sigma$)	2.29 ± 0.08	2.64 ± 0.14	5.33 ± 0.03	10.38 ± 0.56	11.66 ± 0.38	18.46 ± 0.82	29.10 ± 0.31
Repeatibility	96.35%	94.67%	99.37%	94.62%	96.76%	95.57%	98.92%
Deviation	2%	5%	7%	10%	16%	3%	1%
Operator 3 ($\mu \pm \sigma$)	2.64 ± 0.16	3.05 ± 0.27	4.97 ± 0.19	9.25 ± 0.37	9.72 ± 0.19	19.27 ± 0.62	25.89 ± 0.68
Repeatibility	94.06%	91.26%	96.20%	95.99%	98.07%	96.78%	97.35%
Deviation	13%	6%	2%	1%	1%	0%	9%

**Table 3:** Results on calibrated phantoms of Device 3 for the three users.

Device 3							
Gel stiffnes (kPa)	2.24	2.83	4.84	9.12	9.55	19.39	29.46
Operator 1($\mu \pm \sigma$)	2.17 ± 0.39	2.54 ± 0.45	5.14 ± 0.08	8.83 ± 0.19	9.26 ± 1.35	20.59 ± 0.83	28.20 ± 0.47
Repeatability	81.59%	98.2%	98.38%	97.78%	98.53%	95.94%	98.31%
Deviation	2%	7%	4%	2%	2%	4%	3%
Operator 2 ($\mu \pm \sigma$)	1.98 ± 0.02	2.79 ± 0.15	5.19 ± 0.21	9.17 ± 0.17	9.81 ± 0.18	18.61 ± 0.61	27.85 ± 1.69
Repeatability	98.99%	94.48%	95.95%	98.12%	98.11%	96.70%	93.90%
Deviation	8%	1%	5%	0%	2%	3%	4%
Operator 3 ($\mu \pm \sigma$)	2.34 ± 0.12	2.80 ± 0.43	5.77 ± 0.18	9.62 ± 0.15	9.73 ± 0.36	20.59 ± 0.82	27.93 ± 0.13
Repeatability	94.72%	84.46%	96.88%	98.39%	96.25%	95.98%	99.52%
Deviation	3%	1%	14%	4%	1%	4%	4%

### Non-calibrated phantoms

**Figure 8:**
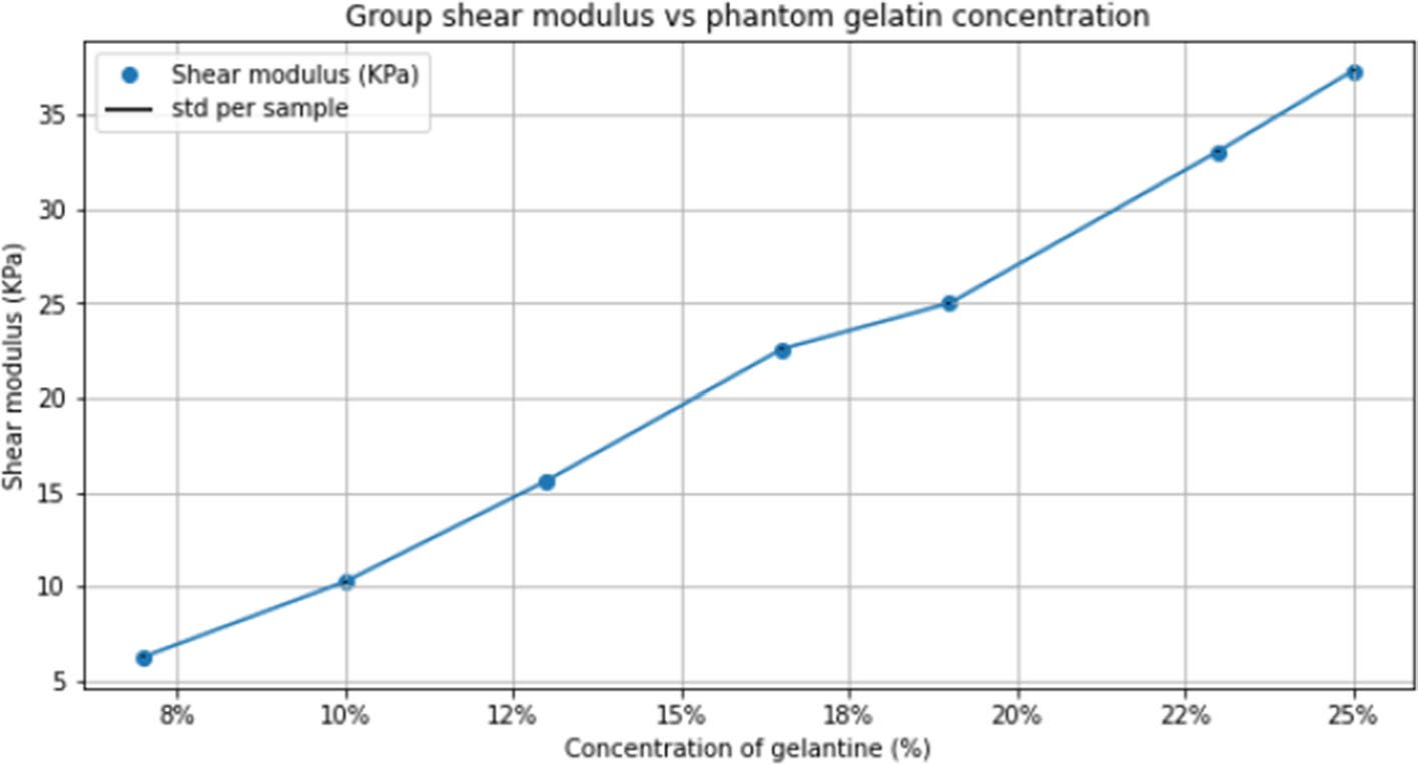
Stiffness elastography results measuring the non-calibrated phantoms.

The stiffness values obtained by SWE for each gelatin concentration are shown in Fig. 8. The stiffness results for each sample are indicated as headings in Tables 4, 5 and 6, wich also report the results of the TWE method in the form of mean ± standard deviation and repeatability, for the Devices 1, 2, and 3, respectively. In this case, only two users performed the measurements because the samples were very deteriorated by time and the results deviated greatly.

**Table 4:** Results on non-calibrated phantoms of Device 1 for the two operators.

Device 1							
Gelatin concentration	7%	10%	13.5%	16.5%	19%	23%	25%
SWE stiffness[kPa]	6.25 ± 0.17	10.24 ± 0.08	15.60 ± 0.03	22.62 ± 0.14	25.00 ± 0.15	33.06 ± 0.1	37.33 ± 0.09
Operator 1($\mu \pm \sigma$)	5.42 ± 0.29	11.60 ± 0.09	14.61 ± 0.66	19.66 ± 0.65	26.65 ± 0.25	26.36 ± 0.40	36.02 ± 0.29
Repeatability	94.62%	99.26%	95.49%	96.70%	99.05%	98.48%	99.19%
Deviation	9%	9%	4%	9%	5%	14%	2%
Operator 2($\mu \pm \sigma$)	5.57 ± 0.07	11.76 ± 0.39	14.69 ± 0.39	18.81 ± 0.18	26.31 ± 0.97	26.08 ± 0.39	36.02 ± 0.29
Repeatability	98.68%	96.69%	97.32%	99.06%	96.33%	98.49%	99.19%
Deviation	8%	10%	4%	12%	4%	15%	2%

**Table 5:** Results on non-calibrated phantoms of Device 2 for the two operators.

Device 2							
Gelatin concentration	7%	10%	13.5%	16.5%	19%	23%	25%
SWE stiffness[kPa]	6.25 ± 0.17	10.24 ± 0.08	15.60 ± 0.03	22.62 ± 0.14	25.00 ± 0.15	33.06 ± 0.1	37.33 ± 0.09
Operator 1($\mu \pm \sigma$)	5.74 ± 0.17	8.06 ± 0.64	10.38 ± 0.80	13.13 ± 0.50	15.70 ± 0.75	22.67 ± 0.74	29.99 ± 0.30
Repeatability	96.96%	91.97%	92.26%	96.19%	95.22%	96.75%	98.99%
Deviation	6%	15%	24%	30%	26%	22%	14%
Operator 2($\mu \pm \sigma$)	5.72 ± 0.19	8.13 ± 0.81	10.26 ± 0.62	13.39 ± 0.23	14.41 ± 0.12	22.63 ± 1.42	29.99 ± 0.31
Repeatability	96.67%	90.01%	94.00%	98.27%	99.15%	93.73%	98.99%
Deviation	6%	15%	24%	29%	30%	22%	14%

**Table 6:** Results on non-calibrated phantoms of Device 3 for the two operators.

Device 3							
Gelatin concentration	7%	10%	13.5%	16.5%	19%	23%	25%
SWE stiffness[kPa]	6.25 ± 0.17	10.24 ± 0.08	15.60 ± 0.03	22.62 ± 0.14	25.00 ± 0.15	33.06 ± 0.1	37.33 ± 0.09
Operator 1($\mu \pm \sigma$)	5.68 ± 0.47	8.45 ± 0.10	14.49 ± 0.80	19.61 ± 0.41	18.74 ± 0.90	26.25 ± 0.88	35.43 ± 0.37
Repeatability	91.67%	98.75%	94.48%	97.88%	95.21%	96.65%	98.97%
Deviation	6%	12%	5%	9%	18%	15%	4%
Operator 2($\mu \pm \sigma$)	5.73 ± 0.28	8.76 ± 0.24	13.84 ± 0.29	18.16 ± 0.53	18.23 ± 0.50	25.69 ± 0.52	35.43 ± 0.37
Repeatability	95.13%	97.28%	97.90%	97.08%	97.25%	97.98%	98.97%
Deviation	6%	10%	8%	14%	19%	16%	4%

### Correlation between techniques

**Figure 9:**
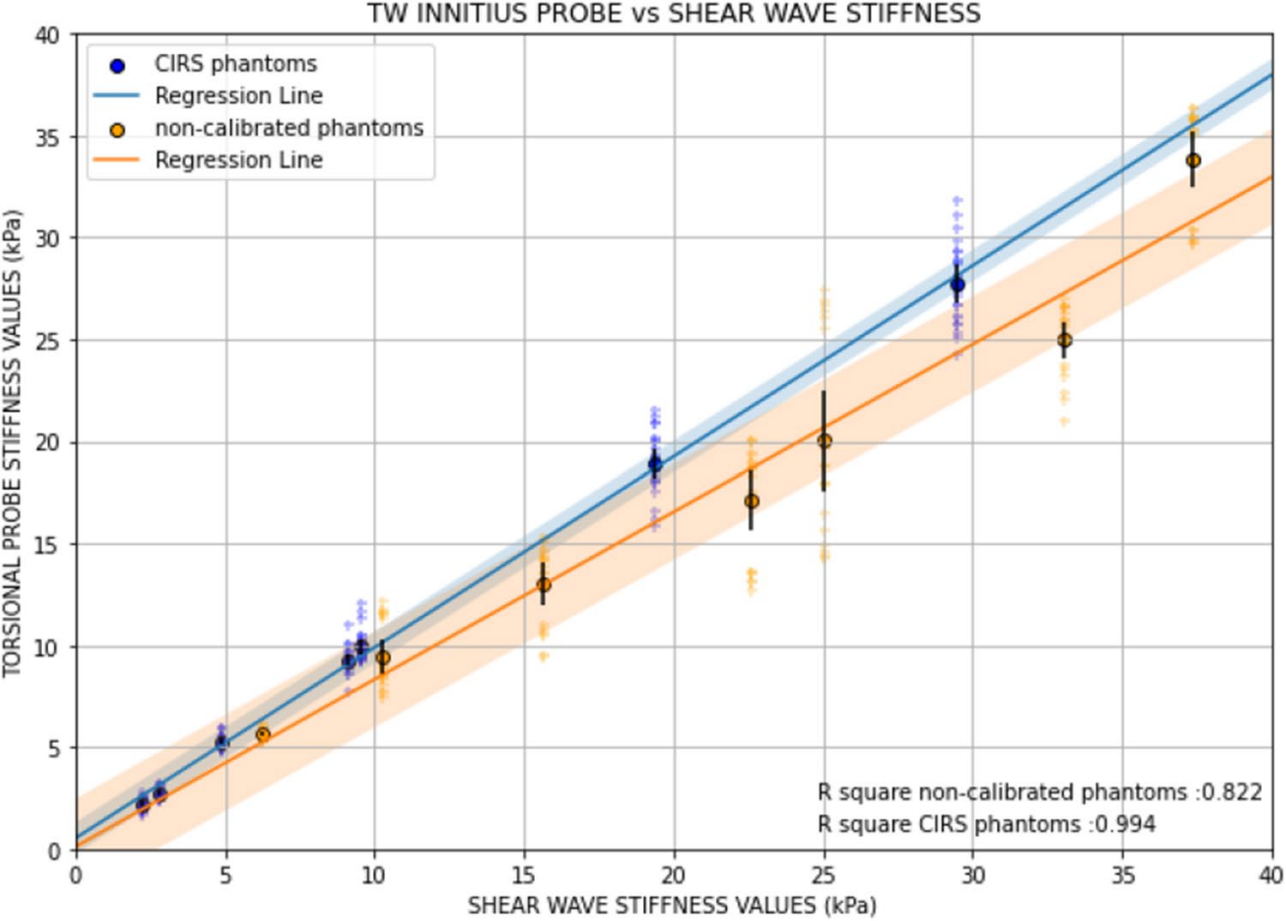
Correlation curves between TWE and SWE methods. The results of the calibrated CIRS phantoms are presented in blue, while the manufactured non-calibrated phantoms are displayed in orange. R square for both test is shown in the figure.

Figure 9 shows the correlation curves between the two techniques. The mean stiffness values and standard deviations have been computed for each phantom, considering both the calibrated and non-calibrated samples. The application of the R2 methodology has facilitated the derivation of a robust quantitative relationship between the measurement methods. The results obtained show a clear relationship between both methods, with R2 > 0.99 on calibrated phantoms, and a lower R2= 0.82 on non-calibrated phantoms. Following previous obtained results it seems that non-calibrated phantoms produce a higher variability in the stiffness values than the calibrated ones. Nevertheless, a clear relationship has been established and both methods show good congruence.

### Reproducibility analysis

Intra and inter-rate correlation between the different devices and operators were calculated for calibrated and non-calibrated phantoms. The ICC results are presented in Table 7.

**Table 7:** Intraclass correlation coefficient (ICC) analysis of inter-device and inter-operator in CIRS samples.

		Intraclass correlation coeff.	95% confidence interval		p value
			Lower bound	Lower bound	
Inter-device	Calibrated phantoms	0.99	0.99	1	9.82e–17
	Non-calibrated phantoms	0.96	0.87	0.99	5.58e–09
Inter-operator	Calibrated phantoms	0.99	0.99	1	3.06e–15
	Non-calibrated phantoms	0.99	0.98	1	4.15e–14

**Figure 10:**
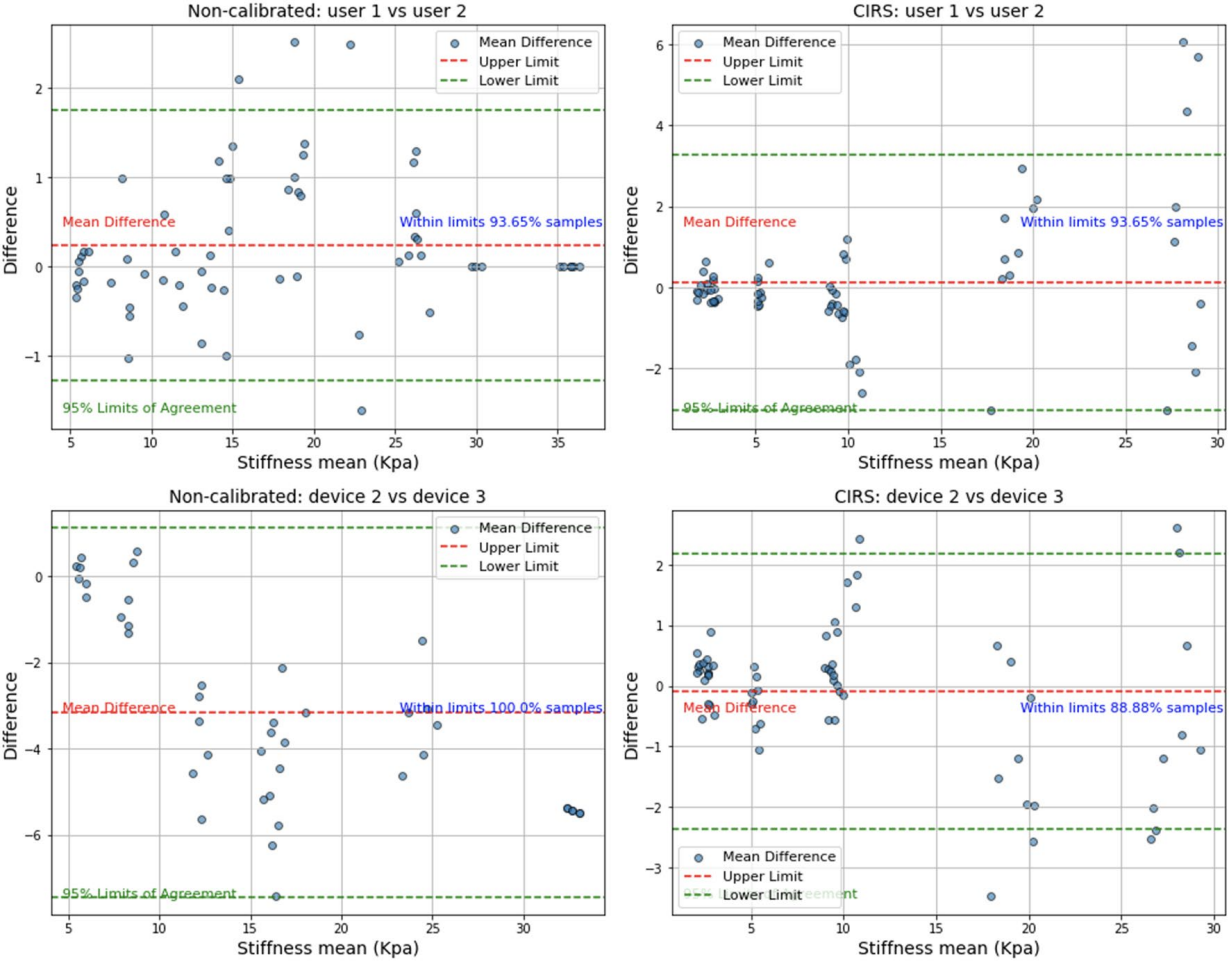
Bland Altman plots: Top left represent the interrelation between users measuring the non-calibrated phantoms while top right refers to the measurements performed over the CIRS (calibrated) phantoms. Bottom left represent the interrelation between different devices measuring the Non-calibrated phantoms while bottom right refers to the measurements performed over the CIRS phantoms.

The obtained results showed excellent reliability between the different operators and devices, being this result statistically significant p-value (< 0.05). Additionally, to assess more visually the similarities and possible bias or trends between different devices and operators, Bland-Altman plots have been generated as shown in Fig. 10 and the Concordance Correlation Coefficient has been calculated between pairs are presented on Table 8.

**Table 8:** Concordance correlation coefficient (CCC) analysis of inter-device and inter-operator in non-calibrated samples.

	CCC between devices			CCC Between users		
	Dev1 vs Dev2	Dev1 vs Dev3	Dev2 vs Dev3	User1 vs User2	User1 vs User3	User2 vs User3
Calibrated	0.984	0.984	0.991	0.983	0.986	0.988
Non-calibrated	0.801	0.940	0.909	0.996	–	–

The obtained results show that there are now visible bias or trends along the different measurements and that most of the obtained samples are in between of the limits of agreement. Besides CCC results showed an excellent agreement with >0.9 values for all combinations of operators and devices.

## Discussion

This study focused on assessing the feasibility of torsional wave technique to quantify the repeatability and consistency of stiffness values compared to standardized technologies in the field. The observed data therefore support that the method can quantify stiffness with high accuracy. The TWE technique, developed by Innitius group, aims to locally measure the mechanical parameters of the cervix, so they can be used as an indicator for assessment in pathologies related with the evolution of cervix consistency.

The proposed Innitius device represents a technological evolution concerning older prototypes since it incorporates the necessary design modifications for application in a real clinical environment^11–15,26,32^. Other similar technologies that quantify tissue consistency may include the Pregnolia device, based on digital palpation^33^ as a basis for the assessment of cervical consistency^20,34^, but this technique is not compared with standard technologies such as SWE.

The CIRS tissue-mimicking phantoms were completely homogeneous samples, as they were manufactured using Zerdine material, a non-flowing water-based, polyacrylamide material which is fully sealed within the phantom housing. The non-calibrated phantoms were manufactured in the laboratory and the homogeneity of the samples were not guaranteed, so it was one of the causes of the differences between techniques. In that case the performance of both techniques was analyzed separately and then analyzed the correlation. Along the comparison of the performance of the different devices measuring the non-calibrated phantoms, the mean difference was not zero and the limits of agreement were not as narrow as they were on the calibrated phantoms. This could be due to the time elapsed between measurements and the degradation of the non-calibrated gels, as different operators all measured each gel with the same probe in order to proceed to the next one until completing the seven gels before switching devices.

In the analysis of the measurement results presented in Tables 1, 2, 3, 4, 5 and Table 6, notable variations were observed between the measurements obtained by different operators. These differences, in some cases, were significant and cannot be ignored. Possible causes of these variations include differences in the technique and experience of the operators, as well as variations in equipment calibration and environmental conditions at the time of the measurements. These discrepancies affect the reliability and reproducibility of our findings, which could bias the conclusions of the study. To mitigate these problems in future research, it is critical to implement standardized training programs for all operators, ensure regular calibration of equipment, and adhere to strict measurement protocols. In addition, where possible, the use of automated measurement systems can help reduce operator-induced variations.

The main limitation of this study is the physical difference between torsional waves and SWE. Both technologies can measure the shear modulus of the samples but it is important to keep in mind that torsional waves obtain the shear modulus from the surface layer of the sample and SWE does it in a more internal layer of the sample. So making a direct comparison assumes that the samples are homogeneous, which is true for calibrated samples but is not insured for non-calibrated samples. On the other hand, laboratory conditions reduce the variability existing in the clinical environment, which will actually be the field of application of the device so it is not advisable to simply assume with the results of this study that robustness and reliability of the device and technology will be maintained.

The proposed stiffness estimation method has probed its intra and inter-correlation reliability obtaining ICC and CCC values over 0.9 for every comparison obtaining p-values below 0.05. According to the obtained results, the inner distribution of the measurements through the different phantoms, non-calibrated and calibrated, did not show any outlier sample. Additionally, comparison with gold standard techniques (SWE) shows that TWE proposed shear modulus estimation are a reliable alternative to stiffness calculation. The design of the study and confidence on certified reference values ensure the credibility and relevance of the findings in stiffness evaluation.

## Conclusions

The presented experimental observations prove that Innitius TW device is capable of consistently and repeatably quantifying the stiffness of elastic materials with high accuracy. In addition, its results have been compared to established techniques with a very high correlation (> 95%), which further supports the results. The advantage of the TWE technique is that it can be integrated into a portable, easy-to-use device, which, as the data show, is not user-dependent and therefore allows stiffness of the tissue to be obtained quickly and easily. Thus, this technology has demonstrated its reliability and robustness compared to other similar techniques and also has great potential for medical application.

The results obtained in this study pave the way to carry out a study, which aims to study the correlation between gestational age and the stiffness of the cervix during pregnancy. A clinical trial has been conducted using the Innitius TW device where the objective was to evaluate the reproducibility and usability of a novel medical device in real environment, the Fine Birth, intended to accurately diagnose the threat of preterm labor by objectively quantifying the pregnant woman’s cervical consistency^35^.

## Data Availability

The datasets used and/or analysed during the current study available from the corresponding author on reasonable request.
